# Architecture and Assembly of the *Bacillus subtilis* Spore Coat

**DOI:** 10.1371/journal.pone.0108560

**Published:** 2014-09-26

**Authors:** Marco Plomp, Alicia Monroe Carroll, Peter Setlow, Alexander J. Malkin

**Affiliations:** 1 Biosciences and Biotechnology Division, Physical and Life Sciences Directorate, Lawrence Livermore National Laboratory, Livermore, California, United States of America; 2 Department of Molecular Biology and Biophysics, University of Connecticut Health Center, Farmington, Connecticut, United States of America; LAAS-CNRS, France

## Abstract

*Bacillus* spores are encased in a multilayer, proteinaceous self-assembled coat structure that assists in protecting the bacterial genome from stresses and consists of at least 70 proteins. The elucidation of *Bacillus* spore coat assembly, architecture, and function is critical to determining mechanisms of spore pathogenesis, environmental resistance, immune response, and physicochemical properties. Recently, genetic, biochemical and microscopy methods have provided new insight into spore coat architecture, assembly, structure and function. However, detailed spore coat architecture and assembly, comprehensive understanding of the proteomic composition of coat layers, and specific roles of coat proteins in coat assembly and their precise localization within the coat remain in question. In this study, atomic force microscopy was used to probe the coat structure of *Bacillus subtilis* wild type and *cotA*, *cotB*, *safA*, *cotH*, *cotO*, *cotE*, *gerE*, and *cotE gerE* spores. This approach provided high-resolution visualization of the various spore coat structures, new insight into the function of specific coat proteins, and enabled the development of a detailed model of spore coat architecture. This model is consistent with a recently reported four-layer coat assembly and further adds several coat layers not reported previously. The coat is organized starting from the outside into an outermost amorphous (crust) layer, a rodlet layer, a honeycomb layer, a fibrous layer, a layer of “nanodot” particles, a multilayer assembly, and finally the undercoat/basement layer. We propose that the assembly of the previously unreported fibrous layer, which we link to the darkly stained outer coat seen by electron microscopy, and the nanodot layer are *cotH*- and *cotE-* dependent and *cotE*-specific respectively. We further propose that the inner coat multilayer structure is crystalline with its apparent two-dimensional (2D) nuclei being the first example of a non-mineral 2D nucleation crystallization pattern in a biological organism.

## Introduction

Spores of bacteria of *Bacillus* species are formed in sporulation and are metabolically dormant and resistant to a large variety of environmental stress factors. While multiple factors contribute to spore resistance, one striking spore feature is the multilayer spore coat that provides protection against many toxic chemicals, as well as digestion by lytic enzymes and being eaten by several types of predatory eukaryotes [1]–[4]. The spore coat is assembled moderately late in sporulation from components synthesized in the mother cell compartment of the sporulating cell, and comprises the outer layers of spores of many *Bacillus* species, although spores of some species contain an outermost exosporium. Spore coat structure and assembly have been best studied in the model spore former *Bacillus subtilis* and ∼70 spore specific proteins have been identified in the spore coat [2], [3], [5], [6]. In addition, a number of these coat proteins undergo covalent modifications including proteolytic cleavage, cross-linking, and tyrosine peroxidation.

The spore coat of *B. subtilis* has drawn attention not only because of its role in spore resistance but also because some coat proteins play significant roles in spore germination. However, much recent work on the spore coat has focused on determining overall spore coat structure as well as the mechanisms involved in the assembly of this large multi-molecular structure. Work to date has indicated that there are at least four coat layers that can be distinguished by electron microscopy (EM) as well as other means – undercoat, inner coat, outer coat, and an outermost glycoprotein layer called the crust [2], [4], [7], [8]. Several of these individual layers also have sublayers, as the inner and outer coats have multiple lamellae. Most of the proteins in these various layers do not have specific roles in spore properties with the exception of a few coat enzymes, and most importantly, proteins that are essential for coat morphogenesis. The morphogenetic proteins include coat proteins such as CotE, CotH, CotO, SafA, and SpoVID, loss of any of which have drastic effects on overall coat architecture, as these proteins direct the assembly of different subsets of proteins into the coat [2], [4], [9]–[11]. In addition, the SpoIIID, GerE and GerR proteins have major effects on the expression of genes encoding coat proteins that are transcribed during sporulation, and this in turn has significant effects on coat properties and morphology [4]–[6].

A variety of studies of the functional repertoire of coat proteins have focused on the determination of the locations of these proteins in the spore coat and their specific roles in spore coat morphogenesis [5], [7], [12]–[15]. These studies have been extended and complemented by studies of direct interactions between various coat proteins, both *in vitro* and *in vivo* [4], [16]–[20]. All of this work has given a picture of the molecular interactions in the spore coat, as well as the dependencies of the assembly of specific proteins into the coat. However, this type of analysis has not yet been complemented by detailed analysis of the structures of the various spore layers. Atomic force microscopy (AFM) has been used to unravel high-resolution structures of the coats of dormant and germinating spores of various *Bacillus* [14], [21]–[30] and *Clostridium* [31] species. However, this analysis has generally been conducted on wild-type spores, with AFM data on only a few mutants lacking specific coat layers. Consequently, in this work we have used high-resolution AFM to analyze the surface structure of spores of wild-type *B. subtilis* spores as well as spores of a variety of mutant strains in order to reveal the surface morphology of various layers of the spore coat. The results from these analyses have provided high-resolution visualization of the various spore coat structures as well as several coat layers not reported previously. This information has allowed the formulation of a model for coat structure and provided further insight into the assembly of the spore coat.

## Materials and Methods

### Strains used in this study

The *B. subtilis* strains used in this study (Table 1) except one are isogenic with the wild-type strain PS832, a prototrophic derivative of strain 168. Preparation of strains by transformation with chromosomal DNA was as described [32].

**Table 1:** *B. subtilis* strains used in this study.

Strain	Genotype	Phenotype^a^	Source or reference^b^
PS832	wild-type		Laboratory stock
PS3394	Δ*cotE*::*tet*	Kan^r^ Tet^r^	[1]
PS3735	Δ*spoVID*::*kan*	Kan^r^	[1]
PS3736	Δ*cotH*::*cat*	Cm^r^	[1]
PS3738	Δ*safA*::*tet*	Tet^r^	[1]
PS4133	Δ*cotB*::*cat*	Cm^r^	DL067→PS832
PS4134	Δ*cotO*::*tet*	Tet^r^	PE250→PS832
DL063	Δ*cotA*::*cat*	Cm^r^	[71]
DL067	Δ*cotB*::*cat*	Cm^r^	[71]
PE250	Δ*cotO*::*tet*	Tet^r^	[87]

a Abbreviations: Cm^r^, chloramphenicol resistant; Kan^r^, kanamycin resistant; Tet^r^, tetracycline resistant.

b DNA from the strain to the left of the arrow was used to transform the strain to the right of the arrow.

### Spore preparation

*B. subtilis* strains were grown at 37°C in Luria-Bertani (LB) [33] medium supplemented with the appropriate antibiotics when necessary. Chloramphenicol was used at a final concentration of 5 mg/liter, kanamycin at a final concentration of 10 mg/liter, and tetracycline at a final concentration of 10 mg/liter.

For spore preparation, *B. subtilis* strains were grown for 3 h in LB medium and then spread on 2× Schaeffer's-glucose medium agar plates without antibiotics [34]. Spores were harvested after incubation at 37°C for 5 d followed by incubation at room temperature for 2 d, and purified as described [34] by brief sonication and repeated washing with distilled water. All spore preparations, except for strain PS3735 (Δ*spoVID*::*kan*) (see below) were free (>98%) of vegetative and sporulating cells and germinated spores as determined by phase-contrast microscopy.

Spores of strain PS3735 (Δ*spoVID*::*kan*) were generally significantly contaminated with germinated spores and these germinated spores were removed by centrifugation in a one-step Histodenz™ (Sigma, St. Louis, MO) gradient. Four samples, each containing ∼3 mg (dry weight) crude spores were suspended in 100 µl of 20% Histodenz™ that was layered on top of 2 ml of 50% Histodenz™ in four Ultra-Clear™ (11×34 mm) centrifuge tubes (Beckman Instruments, Palo Alto, CA) and then centrifuged at 14,000 rpm for 45 min at 20°C in a TLS 55 rotor. After centrifugation, the germinated spores in the supernatant fluid were removed, the pellets containing the dormant spores washed 5 times with 500 µl water and the final pellets were suspended in 500 µl water and combined. These purified spores were free (>98%) from vegetative and sporulating cells as well as germinated spores as determined by phase contrast microscopy.

### Chemical decoating of spores

Spores (∼6 mg dry weight) were decoated as described previously [35], [36]. Briefly, spores were incubated for 90 min at 37°C in 1 ml of 50 mM Tris-HCl (pH 8.0)-8 M urea-10 mM EDTA-1% sodium dodecyl sulfate (SDS)-50 mM dithiothreitol (DTT). After incubation, the spores were centrifuged and the pellets were washed with 1 ml of water 6–10 times.

### Atomic force microscopy

Droplets of ∼2.0 µm of spore suspensions (∼3×10^9^ spores/ml) were deposited on plastic cover slips and incubated for 10 min at room temperature and the sample substrate was carefully rinsed with double-distilled water and allowed to dry. Our prior work with spores of other *Bacillus spp.* [22]–[25] demonstrated that spore morphological and structural attributes were reproduced both for spores analyzed within the same spore preparation and when multiple spore preparations were analyzed. Thus, in this study for each spore strain a single spore batch was analyzed by AFM with ∼50–75 spores being imaged for each spore strain. Detailed experimental procedures for AFM imaging of spores were as described previously [22], [24]. Images were collected using a Nanoscope IV atomic force microscope (Bruker Corporation, Santa Barbara, CA) operated in tapping mode. For rapid low-resolution analysis of spore samples, fast scanning AFM probes (DMASP Micro-Actuated, Bruker Corporation, Santa Barbara, CA) with resonance frequencies of ∼210 kHz were utilized. For high-resolution imaging, SuperSharpSilicon (SSS) AFM probes (NanoWorld Inc, Neuchâtel, Switzerland) with tip radii <2 nm and resonance frequencies of ∼300 kHz were used. Nanoscope software 5.30r3sr3 was used for acquisition and subsequent processing of AFM images. In order to successfully assess both overall low-resolution and high-resolution spore features, raw AFM images typically need to be modified. In particular, the *contrast enhancement* command, which runs a statistical differencing filter on the current image, was typically utilized. This filter can bring all the features of an image to the same height and equalize the contrast among them. This allows all features of an image to be seen simultaneously, and thus a single spore or a group of spores can be imaged at relatively low resolution while visualizing spore coat attributes at high resolution. Heights of spore surface features (i.e. folds, coat layers, etc.) were measured from *height* images using the *section* command, which allows measurements of vertical distance (height), horizontal distance, and the angle between two or more points on the surface. Tapping amplitude, phase and height images were collected simultaneously. Height images allow quantitative height determinations, providing precise measurements of spore surface topography. Amplitude and phase images do not provide height information. While amplitude and phase images provide similar morphological and structural information as do height images, they can often display a greater amount of structural detail and contrast compared with height images, often making them a preferred choice for presentation purposes. The surface roughness of spore surfaces for wild type and *cotE gerE* spores was evaluated as the root mean square (RMS) value R_q_ using AFM height images. R_q_ is the standard deviation of the Z values (height) within the given area and is calculated as: √Σ(Z_i_-Z_ave_)^2^/N, where Z_ave_ is the average of Z values within the given area, Z_i_ is the current Z value, and N is the number of points within the given area. R_q_ was determined for each spore from 4 µm^2^ height images (pixel number −512^2^) of multiple spores using a 400 nm^2^ zoomed in area in the center of the spore. In order to eliminate tilt on the spore surface, prior to the measurement of the roughness, the image was flattened using the third flatten order in the *flatten* command. Step roughness levels were determined by manually digitizing steps' contour from AFM capture images with a plot digitizer (http://plotdigitizer.sourceforge.net/). Once the x and y coordinates of the step contours were obtained, the step perimeter length, *S*, was estimated from the sum of all segment lengths given by *S* = Σ√(Δx^2^+Δy^2^), where the sum is carried over all digitized contour segments. The sinuosity index, which is a measure of step meandering/roughness, is then calculated by taking the ratio of the contour length *S* over the shortest path length between the two end points of the step (straight line). Note, that the value of the sinuosity index ranges from 1 (case of straight line) to infinity (case of a closed loop, where the shortest path length is zero).

## Results

### Surface architecture of wild-type and decoated spore surfaces

**Figure 1:**
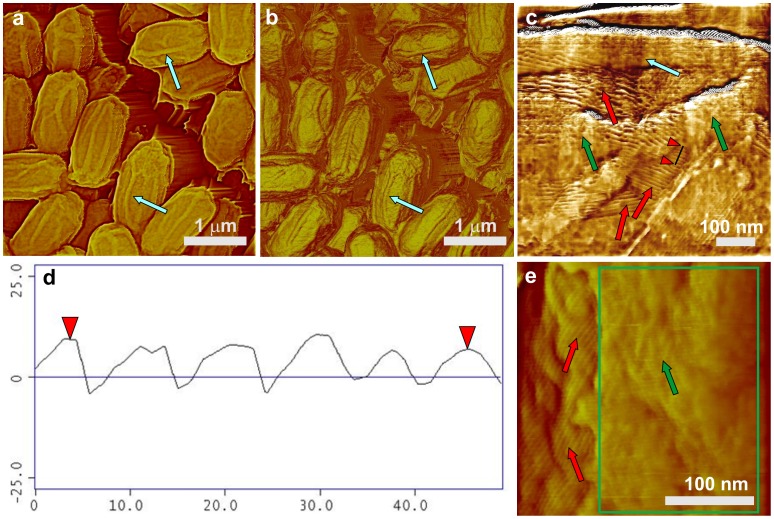
AFM images of *B. subtilis* wild-type spores. (a) Height and (b) phase images of spores with surface ridges (coincidental in both images) extending along the entire length of spores (several surface ridges noted by light blue arrows). (c) High-resolution height image of an area on a surface of a single spore showing surface ridges (light blue arrow), patches of an amorphous outermost layer (green arrows), and a rodlet layer (red arrows) seen beneath the amorphous layer. (d) A cross section line drawn perpendicular to rodlets (indicated with red arrows in (c)) showing a periodicity of ∼8.2 nm. (e) High-resolution height image of an area on the surface of a single spore showing patches of an amorphous outermost layer (green arrow and green rectangle), and a rodlet layer (red arrows) seen beneath the amorphous layer.

As seen previously by AFM [21], [22], the prominent surface features of air-dried wild-type *B. subtilis* spores are surface ridges extending along the long axis of the spore (Fig. 1a,b; light blue arrows). The height of these surface ridges was generally 15–30 nm, occasionally exceeding 40 nm. Similar surface ridges have been observed on spores of *Bacillus anthracis* [29], *Bacillus cereus* [22], [23], *Bacillus atrophaeus* [22], [24], *Bacillus thuringiensis* [22], [23], and *Clostridium novyi* NT [31]. This ridge formation appears to be due to coat folding caused by changes in spore size upon dehydration [22], [24], [30], [37], [38]. RMS roughness R_q_ of wild-type coat surfaces measured as described in the Methods section for 20 spores varied between 3.49 nm to 8.71 nm with an average R_q_ value of ∼5.26 nm.

AFM studies of protozoal-digested coat-defective *B. subtilis* spores [27] showed that the *B. subtilis* spore's outer surface exhibits a thin layer without prominent structural features, which was defined as an amorphous layer (Fig. 1c,e; green arrows). EM of ruthenium red stained *B. subtilis* spores demonstrated the presence of an outermost glycoprotein layer, and it was suggested that this layer is an exosporium that is tightly attached to the coat layer [8]. Later, a combination of EM, fluorescence microscopy, and genetic analysis also demonstrated the existence of this outermost glycoprotein layer that was named the spore crust [7]. Thus the outermost layer revealed by AFM and the crust layer correspond to the same spore layer. The thickness of the outermost amorphous layer in *B. subtilis* spores as measured from AFM images (Fig. 1e) was not uniform and varied between 4–15 nm with an RMS roughness R_q_ of ∼3 nm. Typically, the coverage of surfaces of *B. subtilis* spores with the amorphous layer was not complete, revealing an underlying rodlet layer, seen on all visualized wild-type spores, with a periodicity of ∼7–8.5 nm (Fig. 1c–e; red arrows); note that these rodlets are also seen on the surfaces of the surface ridges. Rodlet structures similar to ones seen in Fig. 1 were previously described in freeze-etching EM [39]–[41] and AFM studies of both fungal [42], [43] and bacterial (*B. atrophaeus*, *B. cereus* and *B. thuringiensis*) [22]–[25] spores, with rodlet structures on *B. atrophaeus*, and *B. cereus* spores exhibiting ∼8 nm periodicity. Note, that depending on sporulation conditions for *B. thuringiensis*, rodlet structures were found either on the spore coat or as extrasporal structures that were present in spore preparations [30].

**Figure 2:**
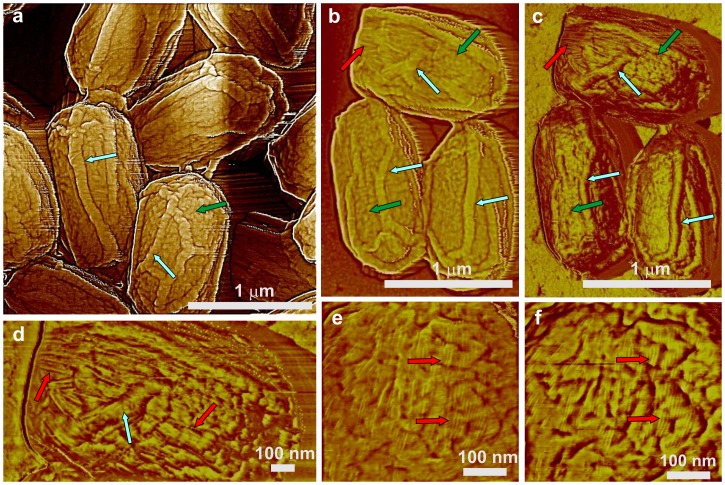
AFM images of decoated *B. subtilis* wild-type spores. Surface ridges extending along the entire length of spores are indicated with light blue arrows in height (a, b) and phase (c, d) images. Patches of rodlet structures are indicated with red arrows in (b–d). The green arrows in (a–c) indicate remnants of the amorphous outermost layer. High resolution height (e) and phase (f) images showing coincidental patches of rodlet structures denoted with red arrows.

In order to remove spores' outer coat, *B. subtilis* spores were chemically decoated with urea-SDS at slightly alkaline pH as described in Methods. The great majority of the proteins removed by this type of treatment have been well characterized in work from a number of laboratories [44]. This treatment partially or completely removed the amorphous layer, and the outer surface of the decoated spores was now comprised primarily of the intact rodlet layer (Fig. 2b–f; red arrows), which was covered in some cases with remnants of the amorphous layer (Fig. 2a–c; green arrows). The 15–30 nm surface ridges were also seen on the air-dried decoated spores, similar to what was seen on intact spores, and again these ridges appear to contain rodlets (Fig. 2a–d; light blue arrows).

### Surface architecture of spores lacking CotA, CotB and SafA

**Figure 3:**
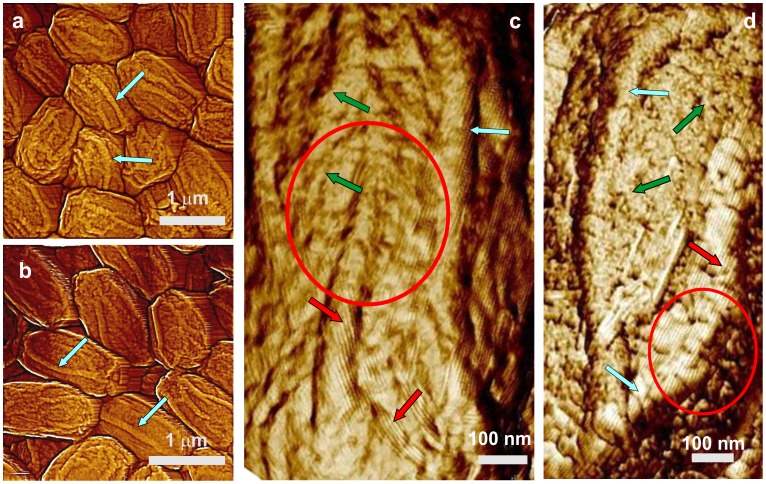
AFM images of *cotA* and *cotB* spores. Height images of *cotA* (a) and *cotB* (b) spores exhibit surface ridges similar to those in wild-type spores (light blue arrows). High-resolution phase images of single *cotA* (c) and *cotB* (d) spores show an irregular outermost amorphous layer (green arrows) as well as underlying rodlets (red arrows). In addition to the amorphous layer and rodlets seen on these spores' outermost surface, a strong undulating topography from a sub-surface layer is also present (red circles). Surface ridges in (c,d) are indicated with light blue arrows.

CotA and CotB are two outer coat proteins that are likely localized on or very near the spore's outer surface [5], [13], [14]. Loss of either of these proteins has no notable effect on spore resistance properties or gross spore coat structure. We found that both *cotA* and *cotB* spore morphologies were indistinguishable from wild-type spores by AFM (Fig. 3), as all *cotA* and *cotB* spores were encased in the outermost amorphous and rodlet layers (Fig. 3c,d; green and red arrows, respectively) and exhibited 20–40 nm thick surface ridges (Fig. 3a–d; light blue arrows). The *cotA* and *cotB* spores also had an undulating surface topography from a subsurface layer (Fig. 3c,d; red circles) that was also seen in wild-type spores (data not shown).

**Figure 4:**
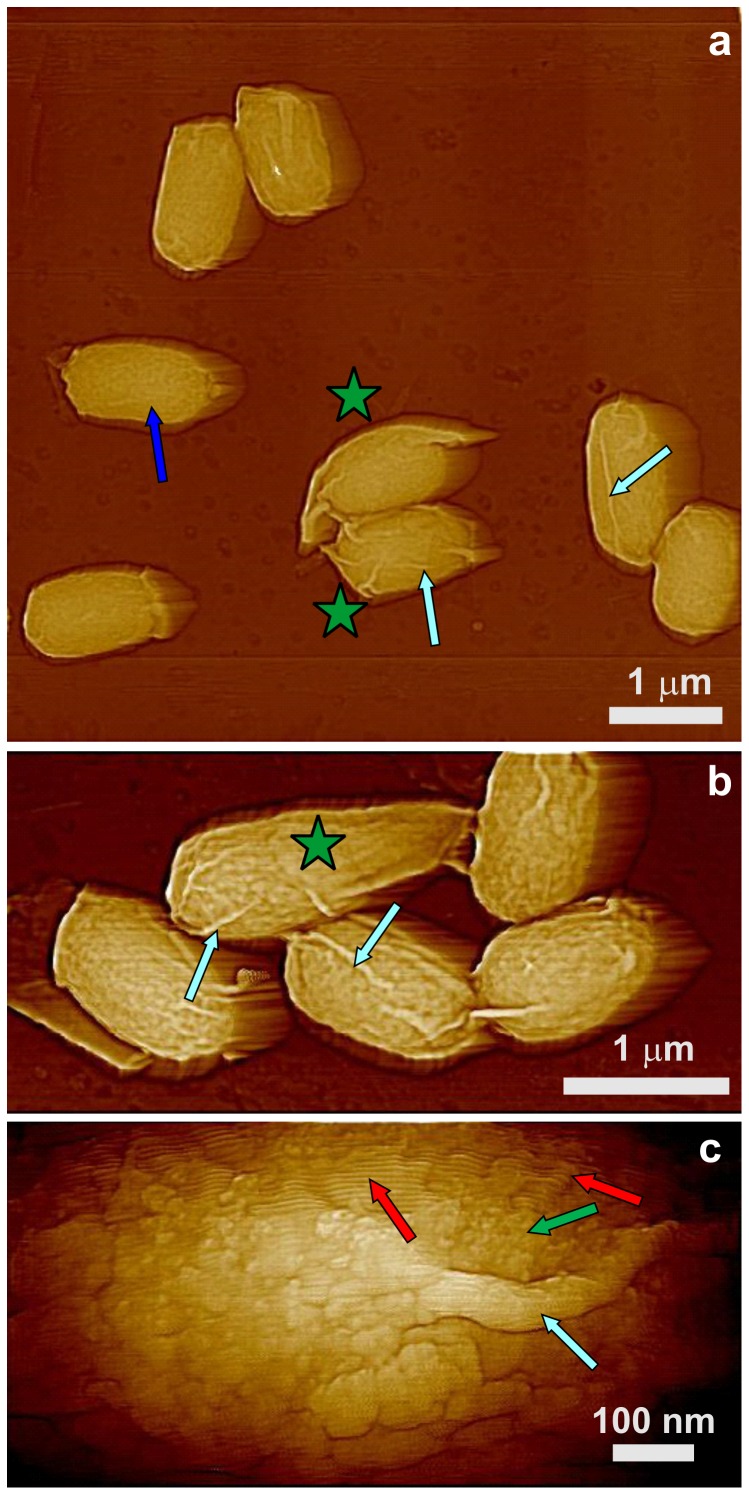
AFM height images of *safA* spores. (a–c) Several surface ridges are indicated with light blue arrows, and in (a) and (b) spores with an oversized sacculus are marked with adjacent green stars. A spore with at most minimal ridges is indicated with a dark blue arrow in (a). In panel (c), two patches of rodlet structure are indicated with red arrows, and a patch of an amorphous layer is indicated with a green arrow.

In contrast to CotA and CotB, SafA plays a significant role in the assembly of at least some components of the spore's outer coat, and much of the coat in *safA* spores does not adhere tightly and can peel off [4], [45]. We observed that the general surface morphology of *safA* spores as seen by AFM (Fig. 4) appears similar to that of wild-type, *cotA* and *cotB* spores, with amorphous and rodlet layers (Fig. 4c; green and red arrows, respectively) forming the outermost s*afA* spores' coat layer. However, the degree of *safA* spore coat folding was different from that in wild-type spores. This resulted in the formation of surface ridges in *safA* spores (Fig. 4a–c; light blue arrows) that appeared shorter (e.g. not running along the whole spore surface as in Fig. 1a) and smaller (ridge heights of 10–20 nm) than in wild-type spores. Furthermore, some *safA* spores had no or minimal surface ridges (Fig. 4a; dark blue arrow), and ∼25% of *safA* spores had an oversized spore coat sacculus that appeared not to be firmly attached to the body of the spore itself (Fig. 4a,b; spores with adjacent green stars, and data not shown), consistent with previous work [44].

### Surface architecture of spores lacking CotO and CotH

**Figure 5:**
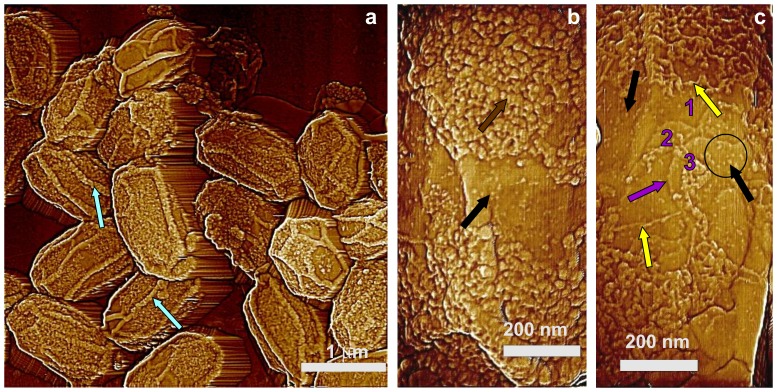
AFM images of *cotO* spores. (a) Height image of spores with surface ridges extending along the entire length of spores (light blue arrows). (b,c) High-resolution height images of areas on surfaces of single spores showing a dense fiber structure forming a granular structure (b; brown arrow) and individual fibers (c; light yellow arrows). In panel (c), three layers (terraces) of inner coat structure are numbered 1, 2, and 3 in purple. Step edges representing boundaries of each layer (one marked with a purple arrow) are visible. In panels (b) and (c), nanodots are marked with black arrows and one area with a high density of nanodots is circled in panel (c).

In addition to SafA, CotO and CotH also play significant roles in outer coat assembly, with perhaps some role in inner coat assembly as well [26], [46]. As seen by AFM (Fig. 5), the outer surface of *cotO* spores was covered either completely or partially by a layer with a grainy appearance (Fig. 5b; brown arrow) and exhibited 15–40 nm thick ridges (Fig. 5a; light blue arrows). The thickness of the grainy layer was 8–20 nm as measured from the AFM images. High-resolution imaging of areas where the grainy structure density was low revealed that this layer actually has a fibrous structure, with the thickness of the thinnest fibers being ∼2–4 nm (Fig. 5c; several fibers indicated with light yellow arrows). Thus, high densities of these fibrous structures appear to have assembled on the inner coat to form a layer that has a granular structure (Fig. 5b,c). Underneath the granular structure, multiple structural layers were observed (Fig. 5c; terraces of 3 consecutive layers numbered 1–3, and the edge of one terrace indicated by a purple arrow), and these terraces were decorated with “nanodot” particles (Fig. 5b,c; groups of nanodots indicated with black arrows, and a circle in 5c). While the heights of some nanodots were as small as 3–4 nm, their typical height was 10–22 nm.

**Figure 6:**
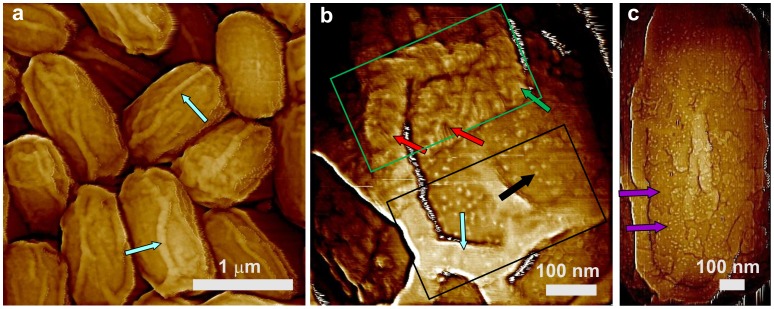
AFM images of *cotH* spores. (a) Height image of spores with surface ridges extending along the entire length of spores (light blue arrows). (b) High-resolution height image of a spore surface area showing the upper surface area (green rectangle) covered with an amorphous layer (green arrow) and rodlets (red arrows). The lower part of the outermost layer-free area (black rectangle) is covered with nanodots (black arrow). One of the surface ridges in (b) is indicated with a light blue arrow. In panel (c), a two–layer inner coat structure (two purple arrows noting the two layers) decorated with nanodots can be seen.

Significant numbers of *cotH* spores (Fig. 6) were also encased in the outermost amorphous and rodlet layers (Fig. 6b; green arrow and red arrows, respectively) and exhibited 15–40 nm thick surface ridges (Fig. 6a,b; light blue arrows). However, 10–15% of *cotH* spores were partially (Fig. 6b) or completely (Fig. 6c) devoid of outer spore coat layers. These *cotH* spores with a defective outer coat exhibited multilayer structures (Fig. 6c; two layers indicated with purple arrows) similar to ones observed on *cotO* spores (Fig. 5b,c). As seen in Fig. 6b,c, these layers again exhibited high densities of nanodots (Fig. 6b; one group of nanodots indicated with a black arrow) similar to ones seen on *cotO* spores (Fig. 5). Nanodot heights appeared smaller and more uniform on *cotH* spores compared with those on *cotO* spores, varying between 2.5–3.5 nm, and none of the *cotH* spores exhibited the fibrous/granular structural layer observed on *cotO* spores.

### Surface architecture of *cotE*, *gerE* and *cotE gerE* spores

**Figure 7:**
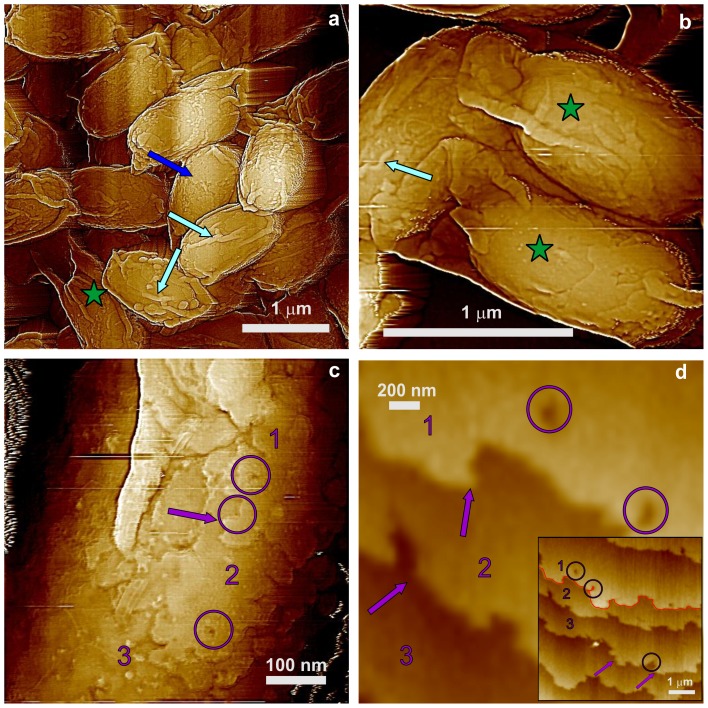
AFM images of *cotE* spores. (a,b) Height images of spores that exhibit surface ridges (light blue arrows), and several spores with an oversize sacculus are labeled with green stars. In (a) a spore with no apparent ridges is indicated with a dark blue arrow. (c) Height image of a multilayer inner coat structure. Three layers are indicated with numbers, and a kink on a step edge is marked with a purple arrow. Several holes in the layered structure are also indicated with purple circles. The hole in the middle circle corresponds to a pinning point on the step. (d) Height image of a multilayer layer structure similar to ones seen in Figs. 5b,c, 6c, and 7c, as seen on the surface of a trypsin crystal. Similar to the spore coat layers in (c), three layers, kinks and several holes are indicated with purple numbers, arrows and circles, respectively. The insert in (d) is a larger area of the crystal surface seen in (d). The same holes and three layers seen in (d) are indicated in the insert. The red line in (d) denotes the step contour, which was utilized for the measurement of the sinuosity index. Panel (d) is reprinted with permission from Plomp M, McPherson A, Larson SB, Malkin AJ (2001). Growth mechanisms and kinetics of trypsin crystallization. J Phys Chem B 105: 542–551. [52]. © (2001) American Chemical Society.

CotE is one of the major morphogenetic proteins in spore coat assembly, and *cotE* spores lack an outer coat and also have alterations in the inner coat layer [2]. AFM images (Fig. 7) revealed that the outermost surface of *cotE* spores is also a multilayer structure, composed of ∼6 nm thick smooth layers (Fig. 7c; three consecutive layers marked as 1–3). These structures are identical to ones observed for *cotO* and *cotH* spores (Fig. 5,6). Note also that: i) the surface of *cotE* spores was devoid of nanodots; ii) the vast majority of *cotE* spores appeared to lack the outermost amorphous and rodlet layers; and iii) *cotE* spores exhibited no granular/fibrous surface structures.

In contrast to wild-type spores, 20–25% of *cotE* spores had no surfaces ridges (Fig. 7a; dark blue arrow) or shorter, thinner ridges (Fig. 7a; light blue arrows) that did not extend across the entire spore surface. The thickness of surface ridges that were seen were only 5–15 nm, less than for surface ridges on intact and decoated wild-type spores (Fig. 1,2). The other interesting morphological feature observed on many *cotE* spores was an oversized spore coat sacculus (Fig. 7a,b; green stars). This was also seen on some *safA* spores (Fig. 4a,b; adjacent green stars), while the wild-type spore coat was always tightly fitted (Fig. 1).

The multilayer outer structure of *cotE* spores (Fig. 7b,c) exhibited step growth patterns similar to those observed on surfaces of inorganic [47], [48] and macromolecular [49]–[52] crystals. An example of similar structures observed on the surface of a growing trypsin crystal [53] is shown in Fig. 7d. Similar patterns were also observed for the inner coat of *C. novyi* NT [31] and *B. anthracis* spores (Plomp and Malkin, unpublished data). As seen in Fig. 7c, layers of structure forming the inner coat of *B. subtili*s spores are similar in morphology to the surface of trypsin crystals (Fig. 7d), with both showing rough steps with many kinks and a number of 5–10 nm (Fig. 7c) and 70–90 nm (Fig. 7d) wide holes (Fig. 7c,d; purple arrows and circles, respectively). The sinuosity index, which is a further measure of the step roughness (see Methods), was estimated for steps on surfaces of *cotE* spores (Fig. 7c) and trypsin crystals (Fig. 7d) as 3.84 and 1.49 respectively. Note that high-resolution AFM observations, which allow at least 1 nm resolution for macromolecular crystalline layers [50], [54], do not result in molecular scale visualization of the molecular packing within the spore coat layers.

**Figure 8:**
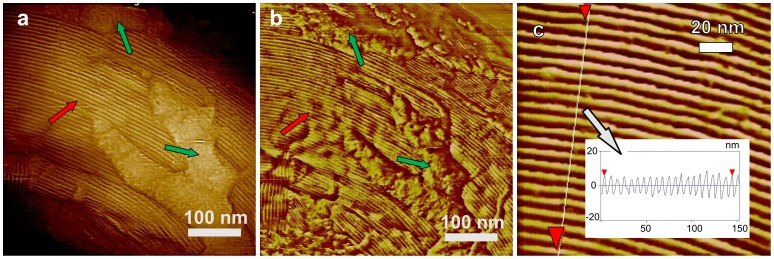
AFM images of *cotE* spores. (a) High-resolution height (a) and phase (b) images of the spore surface showing (coincidental in both images) a rodlet structure (red arrows) covered with patches of an amorphous layer (green arrows). (c) High-resolution height image of the spore surface with an insert with a cross section line drawn perpendicular to rodlets showing the periodicity of ∼7.2 nm.

**Figure 9:**
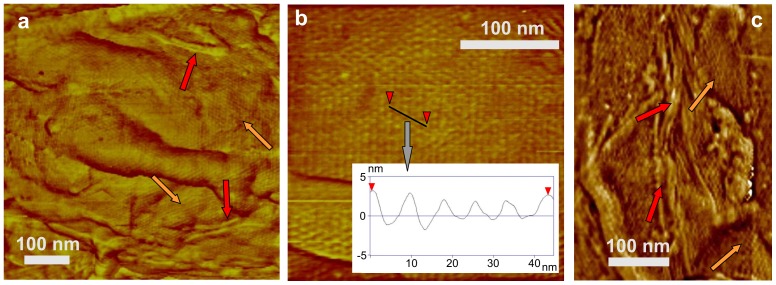
AFM images of *cotE* spores. (a,b) High-resolution height images of the spore surface showing a honeycomb structure (orange arrows in (a)) and patches of rodlets on top seen in (a) (red arrows). The insert in (b) is a cross section line along a honeycomb structure (indicated with a black line and red arrows in (b)) showing a periodicity of ∼8.5 nm. (c) A portion of a loose honeycomb layer (orange arrows) with remnants of rodlet structures (red arrows), which were seen in *cotE* spore preparations.

While most *cotE* spores are encased only in the multilayer coat structure, <5% spores were completely covered by a rodlet layer (Fig. 8a,b; red arrow), with a periodicity of ∼7.2 nm (Fig. 8c; insert). Occasionally, as seen in Fig. 8a,b 4–10 nm thick patches of the outermost amorphous layer were observed atop the rodlet layer of <10% of *cotE* spores (Fig. 8a,b; green arrows). In addition, on <5% of *cotE* spores a honeycomb-like coat layer with a periodicity of ∼8–9 nm was observed atop the inner coat layer (Fig. 9a,b, orange arrow). Note, that loose honeycomb layers with remnants of rodlet structures on top of a honeycomb layer (Fig. 9c, orange and red arrows respectively), were occasionally observed in spore preparations. While >75% of *cotE* spores lacked a complete rodlet layer, these spores still exhibited patches of rodlet structure of different sizes assembled atop the inner spore coat layer (Fig. 9a; red arrows).

**Figure 10:**
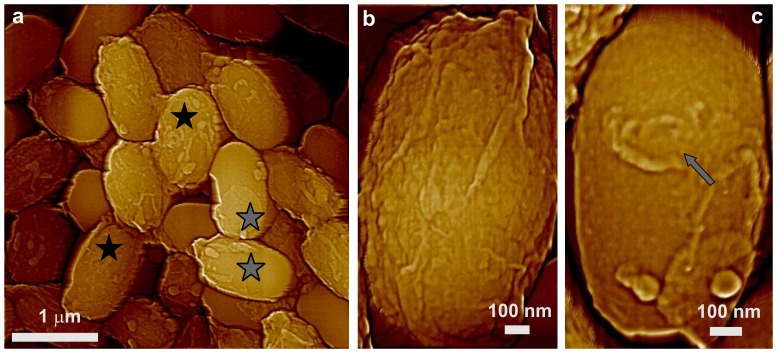
AFM height images of *gerE* spores. (a) *gerE* spores are either completely (black stars) or partially (grey stars) covered with coat material. (b) A spore that is completely encased in the coat material, and (c) a spore with patches of coat material (grey arrow).

In contrast to CotE and other proteins noted above, GerE is a transcription factor that modulates the expression of some coat protein genes late in sporulation, including genes that encode proteins in the insoluble fraction of the spore coat [6]. A *gerE* mutation has drastic effects on overall spore coat structure, as: i) much of the *gerE* spores' coat adheres poorly [55]; and ii) some coat component(s) responsible for the strong X-ray scattering by the spore coat is either absent or misassembled on *gerE* spores, while this X-ray scattering is observed from *cotE* spores [56]. As seen by AFM (Fig. 10), *gerE* spores were devoid of the outer amorphous and rodlet layers, and fibrous structures. Most of these spores were only partially or completely covered by patches of irregular material (Fig. 10a; black stars; Fig. 10b), and 40–45% of *gerE* spores had only patches of this material (Fig. 10a; grey stars; Fig. 10c; grey arrow), with a thin layer of material covering the spore surface (Fig. 10b,c). The thickness of these patches of coat material was ∼6 nm, a value similar to the thickness of the inner coat layers forming the multilayered coat structure (Fig. 5b, 7d).

**Figure 11:**
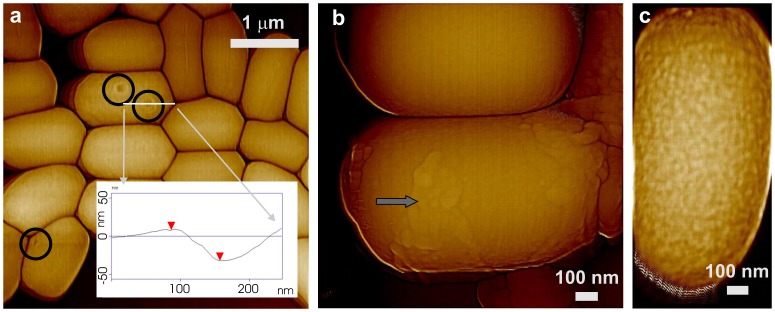
AFM height images of *cotE gerE* spores. (a) Spores which appeared to be devoid of spore coat material. Closely packed spores are more deformed than ones that are not surrounded by other spores. Some spores exhibit 80–100 nm wide and 30–40 nm deep depressions (black circles). The insert in (a) is a cross section line (indicated with a white line) drawn across the ∼100 nm wide depression showing a depth of ∼40 nm. (b) Image showing small patches of coat material (grey arrow) on the spore surface. (c) High-resolution image of a spore devoid of any obvious coat material, and showing a textured outermost surface.

The combination of *cotE* and *gerE* mutations has an even more drastic effect on spore coat structure than either mutation alone, as *cotE gerE* spores are almost completely devoid of a coat (Fig. 11), except for a thin rind of insoluble material [28]. As reported previously [28], with the exception of small numbers of spores which have remnants of coat material (Fig. 11b; grey arrow), >90% of *cotE gerE* spores had none of the spore coat structures described above and their outer surface appeared rather smooth (Fig. 11a,b), although high-resolution imaging revealed a slightly bumpy textured outermost surface (Fig. 11c). The RMS roughness R_q_ of *cotE gerE* spore surfaces measured for 20 spores as described in the Methods section varied between 0.25 nm to 0.49 nm with average R_q_ value of ∼0.38 nm. These severely coat-defective spores also appeared less rigid than intact spores, as *cotE gerE* spores within a closely packed monolayer were more deformed compared to ones with fewer near neighbors (Fig. 11a). Approximately 7% of *cotE gerE* spores also exhibited 80–100 nm wide and 30–40 nm deep depressions (Fig. 11a; black circles), which were also observed on some *gerE* spores (data not shown). Note, that neither *gerE* nor *cotE gerE* spores exhibited surface ridges (Fig. 10,11).

### Surface architecture of *spoVID* spores

**Figure 12:**
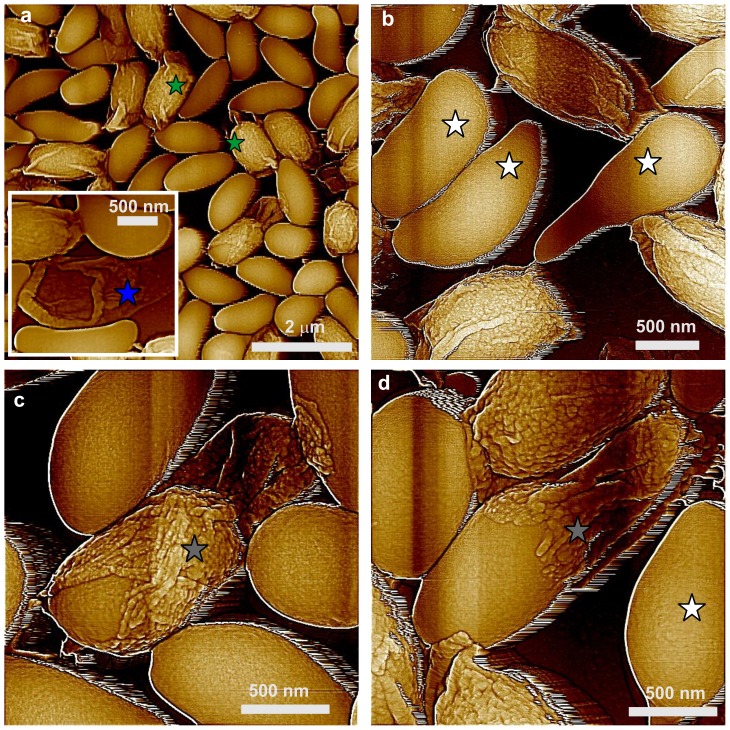
AFM height images of *sspoVID* spores. (a) Many of the *spoVID* spores are devoid of obvious spore coat material, although some *spoVID* spores are encased in loosely fitting coat sacculi (green stars); insert: an empty intact sacculus (blue star) present in a spore preparation. (b,d) Severely deformed spores without any visible coat material are indicated with white stars. (c, d) Spores with partially sloughed off coat sacculii are indicated with grey stars.

SpoVID is another major morphogenetic protein in spore coat assembly. This protein is essential for the adherence and assembly of the coat, and while the peptidoglycan cortex forms relatively normally in *spoVID* spores, the coat largely assembles as swirls in the cytoplasm, giving rise to spores with little coat material [2], [10]. Consequently, the surface architecture of *spoVID* spores is drastically different from that of wild-type spores, as a number of *spoVID* spores were again encased in only loosely fitted coat sacculi (Fig. 12a; green stars). Indeed, for a number of *spoVID* spores, the coat sacculi were partially (Fig. 12 c,d; grey stars) or completely (Fig. 12b,d; white stars) sloughed off, releasing empty sacculi (Fig. 12a, insert; dark blue star) and leaving spores encased in what appeared at lower resolution to be a rather smooth structure (Fig. 12a). Note that the shape of a number of the coatless *spoVID* spores was altered significantly compared either to other mutant spores described above or to *spoVID* spores still encased in coat sacculi. The shape of the coatless *spoVID* spores also varied significantly (Fig. 12b; spores with white stars), sometimes having a shape resembling a bowling pin.

**Figure 13:**
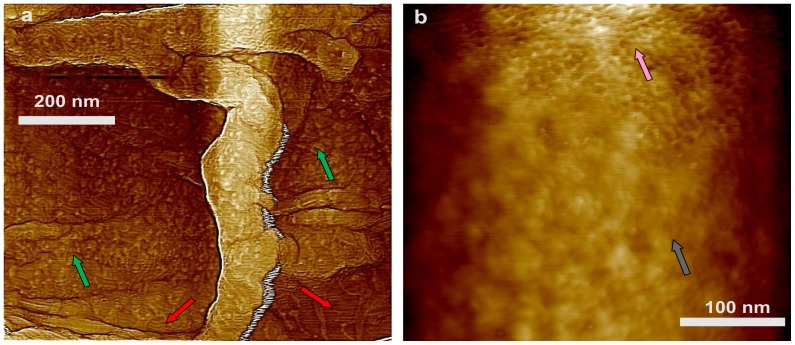
High-resolution AFM height images of *spoVID* spores. (a) External and internal surfaces of the empty coat sacculus in Fig. 12a, insert exhibit morphology similar to that of the outermost wild-type spore layer seen in Fig. 1b. The surface is comprised of rodlets (red arrows) and patches of amorphous material (green arrows). In (b) a pitted layer (pink arrow) is seen beneath a layer of coat material (grey arrow).

The outer and internal surface structures of the coat sacculi released from *spoVID* spores were similar to the outermost surface structure of wild-type spores, as seen in a high-resolution image of a *spoVID* spore sacculus (Fig. 13a), and consisted of rodlet layers (Fig. 13a; red arrows) covered with amorphous material (Fig. 13a; green arrows). As illustrated in Fig. 13b, high-resolution images of the surfaces of the coatless *spoVID* spores revealed a 2–6 nm thick amorphous layer (Fig. 13b; grey arrow) and an underlying pitted surface structure (pink arrow).

## Discussion

### Topography of the outer spore surface

The ridges on the surfaces of air-dried *B. subtilis* spores (Fig. 1a) have been seen previously in EM [39], [40] and AFM [22]–[24], [29], [30] studies of spores of various *Bacillus* species. These ridges have been proposed to form due to the folding of the coat in response to dehydration, likely as a consequence of decreases in spores' internal volume [22], [37], [38]. Indeed, AFM measurements of the morphology of fully hydrated and air-dried spores demonstrate that surface ridges on dehydrated spores mostly disappear or decrease in size upon hydration [22]. Thus, spore coat flexibility can compensate for decreases in spore surface area upon drying by surface folding and ridge formation [22]. Current work demonstrated that this surface folding takes place in the spore coat, since dry *gerE*, *cotE gerE* and *spoVID* spores lacking much of the spore coat exhibit no surface ridges (Fig. 10–12). Note, that while *gerE* and *cotE gerE* spores as well as coat rinds produced by protozoal digestion of spores exhibit some coat material [1], [28] (Fig. 9,10), the amount of this material is either not sufficient or its proteomic composition is not appropriate to form surface ridges. In contrast, the presence of surface ridges on *cotO*, *cotH* and *cotE* spores lacking the amorphous and rodlet layers (Fig. 5–7) indicates again that surface ridge formation takes place within the multilayer spore coat structure.

The spore coats of *B. subtilis* mutants lacking morphogenetic coat proteins as well as chemically decoated wild-type spores have different thickness and composition, and these variations could affect the coat's elastic properties and thus change its folding and surface ridges. However, affecting the outer spore coat architecture by mutation or chemical decoating gave no large changes in spore surface ridge parameters or patterns. These results suggest that formation of spore surface ridges originates within multilayer coat structures, which are relatively unaffected by loss of some coat proteins, with the amorphous, rodlet and fibrous layers only following the ridge-associated topography. Our data showing pronounced changes in the surface folding of *safA* spores (Fig. 4) may indicate that these spores' multilayer coat structure is either thinner or more flexible than in wild-type, decoated wild-type, *cotA*, *cotB*, *cotH*, and *cotO* spores (Fig. 1–,5,6). However, *cotE* spore coats with lower levels of surface folding typically have the same number of layers as do *cotH* and *cotO* spore coats. Perhaps the decreased surface folding of *cotE* spore coats is due to changes in the elastic properties of inner coat layers because one or more inner coat proteins are not assembled in *cotE* spores. Note, that the wide range of surface ridge parameters and folding patterns observed with spores of different species [22]–[24] and isogenic strains (this work) makes it problematic to assign these parameters as spore species-specific structural attributes.

### Spore coat architecture

**Figure 14:**
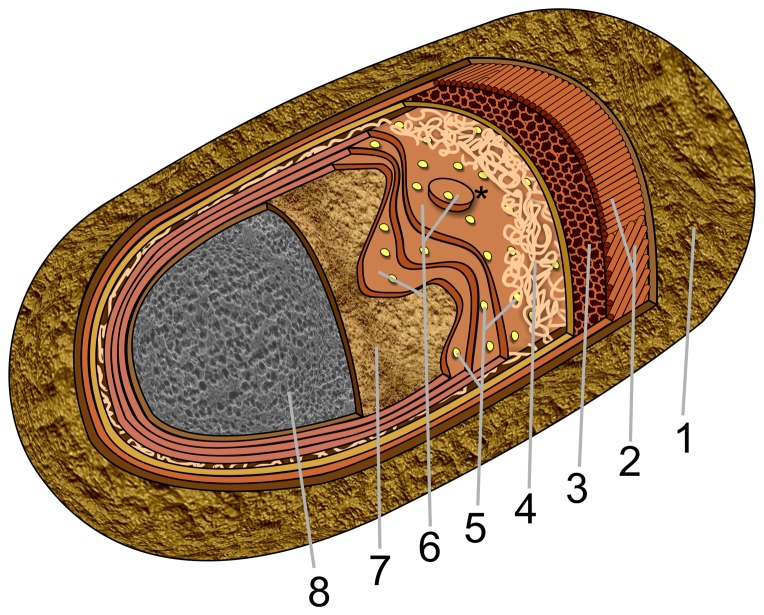
Model of the spore coat architecture of a single *B. subtilis* spore. The layers of the spore coat and the cortex are depicted as: (1) an outermost amorphous layer (the crust); (2) the rodlet layer; (3) the honeycomb layer; (4) the fibrous/granular layer, (5) the nanodot layer on top of a multilayer structure (6) ((with a 2D nucleus (indicated with *) seen on the upper layer)); and the basement layer (7), which is on the top of the cortex's outer pitted surface (8). Structural features of spore coat layers are not shown to scale.

In addition to providing information on spore surface topography, AFM images allowed construction of a detailed model of coat architecture (Fig. 14). In this model starting from the outside, the coat consists of an amorphous layer/crust, a rodlet layer, a honeycomb layer, a fibrous layer, a nanodot particle layer, the multilayer assembly, and the undercoat/basement layer just above the pitted surface, which we tentatively assign as the spore cortex.

The existence of an outermost tightly fitting spore layer was initially reported in thin section EM images of *B. subtilis* spores treated with a reducing agent [57]. It was suggested that this layer is an exosporium-like structure, which in EM images of untreated spores is usually indistinguishable from the darkly stained outer spore coat. While the amorphous spore coat layer reported here (Fig. 1) could correspond to this outer coat structure, it does not resemble an exosporium, as this outer layer has no paracrystalline basal layer typical of the exosporium of spores of the *B. cereus* group [41], [58]. Rather the amorphous layer likely corresponds to the outer crust layer of *B. subtilis* spores that stains with ruthenium red and is glycoprotein-rich [7], [8]. Patches of an outermost amorphous layer are also observed in AFM studies of *B. atrophaeus* spores (Plomp and Malkin, unpublished data).

Rodlet structures, similar to ones seen in Fig. 1b,c, were previously described on the outer surface of a diverse set of microorganisms (see [59]), including Gram-negative bacteria and various fungi. The fungal rodlet layers were resistant to treatment by detergents, organic solvents, enzymes, alkali and mild acids [60], [61], and the structural proteins hydrophobins [62], [63] and chaplins [64] were integral components of fungal rodlet structures. In several cases, these rodlets had a cross β-structure similar to that in the amyloid fibrils [64] associated with several neurodegenerative diseases [65]. The amyloid-like rodlet fibrils forming microbial outer surface layers appear to play important roles in attachment, dispersal and pathogenesis [59].

Rodlet structures were also reported in EM [38]–[40] and AFM studies [22]–[24], [30] of spores of several *Bacillus* species, although the proteins that form these rodlet structures are not known. The structural similarities between *B. atrophaeus* rodlets seen during their germination-induced disassembly [25] and amyloid rodlets found on surfaces of fungi and bacteria suggest that *B. subtilis* coat rodlets may also be amyloids. However, full understanding of the function of rodlet structures in spores awaits elucidation. Interestingly, micro-etch pits form in the rodlet layer early in spore germination [25], and these could facilitate access of degradative enzymes to their targets in an otherwise tightly packed coat. Characterization of the strength and mechanical stiffness of individual amyloid fibrils of insulin reveals that these parameters are similar to those of steel and silk [66]. Thus, the spore coat' rodlet layer could play a role in protecting spores from mechanical stress, and a combination of rodlet and amorphous structures could provide spores with a wide range of physicochemical properties. Indeed, the existence of both hydrophobic (rodlets) and hydrophilic (glycoproteins) structures on the outermost layer might enable spores' successful dissemination as both as air-born and fully hydrated particles.

Current results indicate that the assembly of the outermost coat layer does not require CotA and CotB, two of the most abundant outer coat proteins [2], [14], [67]–[71]. While interactions between CotB and CotG are critical in guiding assembly of the outer coat layer, no coat assembly defect has been observed in *cotA* or *cotB* mutants [69], [70]. In addition, *cotA* and *cotB* mutations have no effects on spore lysozyme resistance, germination [2] or surface appearance (Fig. 3), and CotB is absent from *cotH* spores [72] yet the outermost coat structure of CotH spores is similar to that of wild-type spores (Fig. 6), including both the rodlet and amorphous layers. Thus, neither CotA nor CotB appear to play important roles in directing the assembly of spores' outer layers. The similar surface ridges on *cotA* and *cotB* spores further suggests that loss of these proteins does not significantly alter the elastic properties of the spore coat.

Loss of SafA also does not affect the high-resolution architecture of spores' amorphous and rodlet layers (Fig. 4), consistent with *safA* spores' lysozyme resistance [2], [45]. However, SafA plays an important role in coat assembly, as in many *safA* spores the coat is loosely attached to the spore (Fig. 4). SafA is localized in the spore cortex near the inner coat, and SafA may help associate the spore cortex and coat [2], [73]. The absence of surface ridges on a large portion of *safA* spores, along with relatively thinner existing surface ridges, also suggest that *safA* spores' coat is thinner and/or more flexible. This is consistent with EM analyses that indicate the *safA* spore coat often has 1–2 layers instead of the typical 3–5 layers [45].

### Inner coat structures

In *B. cereus* [22] and *B. atrophaeu*s spores [25] the coat's rodlet layer is underlain by a honeycomb structure also observed in *B. subtilis* spores (Fig. 9). Since disordered microporous inorganic substrates can effectively initiate three-dimensional protein crystallization [74], perhaps the spore coat's honeycomb structure represents a biological example of a microporous matrix that facilitates the ordered self-assembly of the coat's rodlet structure. Note that the 8–9 nm periodicity of the *B. subtilis* coat honeycomb layer is similar to periodicities of honeycomb structures for *B. cereus*, *B. thuringiensis* [22] and *C. novyi* NT [31] spores. This indicates that molecular dimensions of proteins forming these honeycomb structures are similar for different bacterial species, and the molecular composition of the honeycomb layer in different bacterial species may thus be similar.

Studies of *cotO*, *cotH* and *cotE* spores revealed consecutive structural layers of granular/fibrous material (*cotO* spores; Fig. 5), nanodots (*cotO* and *cotH* spores; Fig. 5, 6), and multilayer structures (*cotO*, *cotH* and c*otE* spores; Fig. 5–7). While spores of these mutants had the multilayer structure, only *cotO* spores retained the granular/fibrous structure and *cotE* spores lacked the nanodot layer. We propose that the granular/fibrous layer represents an outer spore coat layer that appears as a darkly stained irregular layer in EM images [69]. The thickness of this outer coat layer varies significantly in EM images on both the same spore and between spores, consistent with the range of granular/fibrous layer thickness observed on *cotO* spores.

On wild-type spores and spores of some mutants lacking specific coat proteins (i.e. *cotA* and *cotB*), the grainy/fibrous outer coat layer was largely obscured by the rodlet and amorphous layers. However, the force exerted by the AFM probe tip on the outermost spore layer allows visualization of underlying structures, as in the AFM visualization of a cytoskeleton beneath a cellular plasma membrane [75]. AFM phase imaging can probe micromechanical properties of sample materials (e.g. viscoelasticity) [76] and map surface inhomogeneity of these properties. Furthermore, when mechanical properties of two layers are significantly different, phase imaging can provide structural information on layers beneath the topmost layer [77]. Thus an irregular grainy layer can often be seen in AFM phase images beneath the outer rodlet structure (Fig. 3c,d), and we suggest that this underlying layer corresponds to a grainy/fibrous outer coat layer (Fig. 5, 414). Note, that an undulating surface morphology similar to that seen on *cotA* and *cotB* spores (Fig. 3c,d) was also observed on the surface of wild-type spores (data not shown).

Typically, multilayer structures on *cotO*, *cotH* and *cotE* spores contained 3–5 layers, consistent with the appearance of the lightly staining lamellar inner coat of *B. subtilis* spores seen by EM [68], and thus these multilayer structures may correspond to the *B. subtilis* spores' inner coat. We further suggest that the nanodots between the outer and inner coat layers but absent on *cotE* spores, might be CotE molecules that facilitate the assembly of the grainy/granular outer coat layer. The height of the smallest nanodots seen on *cotH* spores was ∼3 nm, consistent with CotE's mol wt of 20.9 kDa [2], and this suggestion is consistent with current models of *B. subtilis* spore coat assembly that have CotE positioned between the inner and outer coat layers [2], [26]. However, these nanodots could also be small coat protein aggregates, and further experiments, perhaps using AFM-based immunolabeling techniques (29), will be needed to identify the protein(s) forming the nanodots.

The *cotO* spores have no amorphous or rodlet layers, which could explain the partial lysozyme sensitivity of *cotO* spores [26]. However, the presence of these outer layers on the majority of *cotH* spores (Fig. 6) is consistent with their relatively normal lysozyme resistance [1]. The outer coat of *cotO* spores often appears disorganized and missing in EM thin sections [26] and is generally indistinguishable from that of *cotH* spores. CotO and CotH are suggested to be localized below the coat surface [13], [26] and to participate in a late phase of coat assembly. However, our AFM analyses showed pronounced differences between *cotO* and *cotH* spore coats. In particular, CotO plays a critical role in the assembly of the amorphous and rodlet layers, while assembly of the fibrous outer coat requires CotH and CotE. AFM studies also indicated that these proteins play a role in assembly of the coat's amorphous and rodlet layers, consistent with biochemical, genetic and EM studies [26], [46], [72]. It has been suggested [26] that CotO and CotH also play an important role in inhibiting the tendency of outer coat protein layers to stack up resulting in the polymerization of the coat layers into closed shells. However, AFM demonstrates that *cotO* and *cotH* spore coats self-assemble to form contiguous shells rather than disorganized coats. At the same time, many *cotE* spores exhibited only a loose coat sacculus (Fig. 7), indicating that CotE plays an important role in the assembly of the inner coat and/or its attachment to the cortex as noted above.

The crucial role for CotE and GerE in proper coat assembly was further highlighted by the AFM of *gerE* and *cotE gerE* spores. First, loss of *gerE* prevented formation of the outer coat, rodlet, and amorphous layers. Second, while most *gerE* spores are encased in a loose structure formed by what appeared to be patches of the inner coat (Fig. 10), these structures do not resemble the inner coat multilayer structures described above. The *cotE gerE* spores were devoid of the amorphous and rodlet layers, and both complete inner and outer coats, and these spores' surface exhibited some roughness (Fig. 11). This surface likely corresponds to the basement/undercoat layer [4]. Thus, both CotE and GerE are crucial in proper assembly of the inner coat. Note, also that *cotE gerE* spores are less rigid than *cotE* or *gerE* spores. This increased deformability is due either to the loss of the inner coat or a role for CotE in the assembly and elastic properties of the basement layer. The nature of the 80–100 nm wide and 30–40 nm deep depressions seen in Fig. 11a is unclear, but we speculate that these holes may facilitate germinant access to the spore inner membrane, and are perhaps associated with the GerP proteins important in germinant movement through spores' outer layers [78].

Another coat protein important for proper spore coat assembly and attachment to the cortex is SpoVID, as a large percentage of *spoVID* spores lacked obvious coat structures, with some encased in a misassembled sacculus composed of amorphous and rodlet structures (Fig. 12). The thickness of the sacculi walls varied between 15–30 nm, indicating that the sacculi could contain coat material in addition to the rodlet and amorphous layers (Fig. 13a). Note that none of the *spoVID* spores visualized in this study exhibited the multilayer inner coat structures seen on *cotO*, *cotH* and *cotE* spores indicating that the inner coat is absent on *spoVID* spores. Most *spoVID* sacculi were only loosely attached to the spore body and were partially sloughed off, exposing a relatively smooth spore surface (Fig. 12). These AFM data are consistent with observations of swirls of spore coat in *spoVID* mother cells [10] (Fig. 12a, insert) and that SpoVID is required for the stable attachment of the coat. High-resolution imaging of the surface of *spoVID* spores indicated the existence of two prominent layers (Fig. 13b). One layer (13b; square) could correspond to the basement layer [4] and a pitted layer (Fig. 13b; black arrow) could correspond to either a subbasement coat layer or the cortex. Note, that *spoVID* spores lacking sacculi exhibit very high deformability (Fig. 12).

During wild-type *B. subtilis* sporulation, proteins forming honeycomb and rodlet coat layers self-assemble on the outer spore coat layer. Based on AFM results with *cotE* spores, the complete outer coat layer is not essential for formation of patches of the honeycomb and rodlet coat layers. Thus, the underlying integument is not crucial for assembly of the rodlet and honeycomb layers. Proteins that form honeycomb and rodlet spore coat structures must therefore be present during *cotE* spore formation, and self-assemble on the outer spore surface producing amorphous and rodlet layers (Fig. 8). Indeed, during *B. thuringiensis* sporulation rodlet proteins can self-assemble on the underlying spore coat, or in either the mother cell cytoplasm or the sporulation medium [23]. Hydrophobins, which form fungal rodlet layers, also self-assemble into rodlet fibrils *in vitro* (for review see [59]).

**Figure 15:**
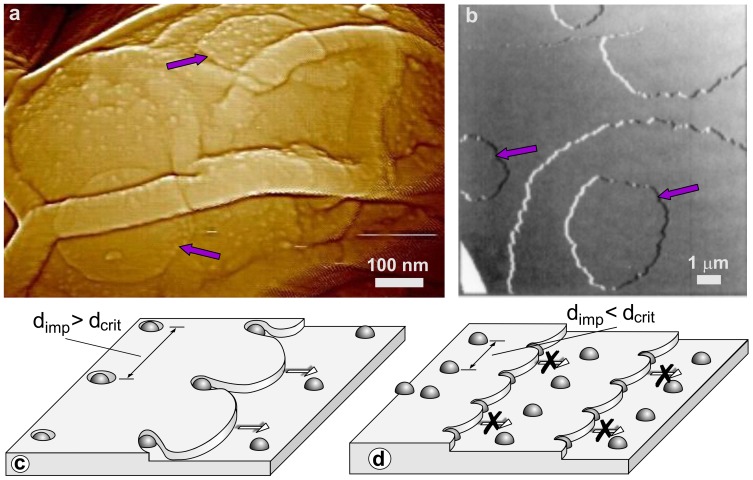
2D nucleation and growth of inner spore coat layers. Panel (a) shows two putative 2D nuclei (purple arrows) on the inner coat surface of a *cotO* spore. Panel (b) shows 2D nuclei (purple arrows) on the surface of a satellite tobacco mosaic virus (STMV) crystal. This illustration is reproduced with permission from Malkin AJ, Kuznetsov YuG, Land TA, DeYoreo JJ, McPherson A (1995) Mechanisms of growth for protein and virus crystals. Nature Struct Biol. 2: 956–959 [48]. © (1995) Nature Publishing Group. (c) At a relatively small impurity (indicated as small balls) density, the average impurity distance *d*_imp_ is larger than *d*_crit_ and steps are able to advance. (d) At higher impurity densities, *d*_imp_<*d*_crit_, the curvature of step segments between impurities increases and steps are halted. Panels (c) and (d) are reproduced, with permission from Plomp M, McPherson A, Malkin AJ (2003). Repair of impurity-poisoned protein crystal surfaces. Proteins: Struct, Function, Bioinform 50: 486–495 [82]. © (2003) John Wiley and Sons.

The multilayer structure forming the inner coat of *B. subtilis* spores exhibits patterns similar to ones described for the inner coats of spores of *C. novyi* NT [31] and *B. anthracis* (Plomp and Malkin, unpublished data). These patterns are also similar to those observed on surfaces of inorganic and macromolecular crystals. In addition to growth steps, these patterns include two-dimensional (2D) nuclei and screw dislocations that are major growth sources of inorganic, organic, and macromolecular crystals [79]. The presence of these growth patterns plus the smooth appearances of coat layers strongly point to a crystalline nature [79] of *B. subtilis* inner coat layers. While no screw dislocation sources similar to ones observed on the *C. novyi* NT inner spore coat [31] were seen on *B. subtilis* spores, on some spores with a low density of the grainy/fibrous outer layer, circular 2D nuclei were observed on the inner coat (Fig. 15a; dark blue arrows). This indicates that *B. subtilis* spores could represent the first case of non-mineral 2D nucleation growth patterns in a biological organism.

The observations above strongly suggest that assembly of inner spore coat layers proceeds by formation of 2D nuclei and their subsequent growth, similar to the birth-and-spread growth mechanism of conventional and macromolecular crystals [51], [79]. In this model, 2D crystal growth takes place by generation and subsequent spread of 2D nuclei that provide a new crystalline layer on crystalline surfaces. Subsequent formation and growth of new 2D nuclei on this layer result in the formation of a new crystalline layer. An example of such growth, showing 2D nuclei on the surface of a crystal of satellite tobacco mosaic virus that are similar to ones seen in Fig. 15a, is presented in Fig. 15b (dark blue arrows). Typically, 2D growth takes place at high supersaturation (e.g. protein and precipitant concentrations used in macromolecular crystallization) [51], [52], [79], suggesting that relatively high concentrations of inner coat protein(s) are present during *B. subtilis* sporulation.

Step edges seen on the inner coat of *B. subtili*s spores showed significant roughness with many kinks (Fig. 7b), suggesting that formation of the inner coat was strongly affected by impurities. Similar patterns have been described for a wide range of crystalline surfaces (illustrated in Fig. 7d), where adsorption of impurities (ones present in solution), but not forming a layer at the step terraces and edges results in step roughening and cessation of growth [80]–[82]. Indeed the roughness and sinuosity of step edges on the inner coat of *B. subtilis* spores (Fig. 7c) are higher than observed for step edges on the surface of the trypsin crystal (Fig. 7d). This may indicate [80]–[83] that higher levels of impurities are adsorbed on the inner coat surface of *B. subtilis* spores compared to ones on the surface of trypsin crystals. Growth steps stop at sites of contact with impurity particles (indicated as small balls in Fig. 15c,d) that are adsorbed to the surface. However, portions of steps between neighboring impurity particles continue to grow, resulting in pinning of growth steps (Fig. 15c) as seen in Fig. 7c,d. Step advancement ceases (Fig. 15d) when at increased impurities' concentration, the distance between impurities/pinning points *d_imp_* becomes smaller than the diameter of critical nuclei *d_c_* necessary for step advancement [80]. One interesting feature of the inner coat is a number of ∼5–10 nm holes (Fig. 7c), which may indicate locations of clusters of impurities [53], [83]. Note, that in general the size of such holes is a function of the size of impurities or their clusters adsorbed on the surface. As described for a number of systems [80]–[83], these clusters of impurities may be responsible for pinning the advancement and cessation of growth of spore coat layers observed in Fig. 7c. Alternatively, such holes that were also observed on inner coat layers of *C. novyi* NT spores [31] and *B. anthracis* spores (Plomp and Malkin, unpublished data) could be an intrinsic feature of spore inner coat layers having a particular function. These results, combined with prior observation of screw dislocations on the inner coat of *C. novyi* NT spores [31], strongly suggest that inner spore coat assembly is governed by two crystallization mechanisms – growth on dislocations and 2D nucleation. These observations suggest that while spore coat proteins are produced enzymatically [84], the assembly of these proteins into coat layers may be a self-assembly process similar to crystallization, and may be influenced by the sporulation conditions (protein and salt concentrations, pH, temperature, impurities) when these proteins assemble.

The lack of high-resolution crystalline lattice structures of the *B. subtilis* inner coat layers is similar to prior observations of *C. novyi* NT [31] and *B. anthracis* inner spore coat layers (Plomp and Malkin, unpublished data). It was suggested that proteins forming the *C. novyi* NT inner coat layers [31] are not globular, but rather peptides ‘standing upright’ in the layers, similar to peptide arrangements found in several organic crystals [85], [86]. This hypothesis was based on the fact that for globular proteins, the ∼6 nm height of the inner spore coat layers would not be considerably different in either perpendicular or lateral unit cell parameters, with the latter being amenable for AFM visualization [54]. Based on the lack of molecular scale AFM resolution of the crystalline lattice forming the *B. subtili*s inner coat layer, it is reasonable to suggest that proteins forming the inner coat might be also “standing upright” peptides [31], [85], [86].

In conclusion, the results presented in this communication provide further understanding of the structure and assembly of the *B. subtilis* spore coat. Furthermore, morphological and structural attributes of *B. subtilis* spores described here could thus serve as a baseline for future studies of effects of sporulation conditions on these structures. In addition, the similarities of some of the new findings with *B. subtilis* spores to findings with spores of *C. novyi* NT and other *Bacillus* species, suggest that the coat structure proposed in this work may generally be similar for spores of all of these species. While there is extensive knowledge of the individual proteins in the spore coat, as well as their location and assembly, there is much less knowledge of precise coat structure. In particular, the new high-resolution AFM studies have identified a number of new coat structural features, including the nanodots, the fibrous layer, and the terraced multilayer inner spore coat. Based on these results, we propose that the amorphous/crust layer and rodlets form the outermost spore structure, the fibrous layer and multilayer structure correspond to the outer coat and the inner coat respectively, with honeycomb and nanodot structures sandwiched between the outermost layer and the inner coat and the inner and the outer coats respectively.

Note, that high-resolution studies of fully hydrated *B. atrophaeus* [22], [25] and *Clostridium novyi* NT spores [31] demonstrated that rodlet, honeycomb, and inner coat layer structures, similar to ones described here for *B. subtilis*, maintained the same patterns, lattice periodicities, and step heights as seen on air-dried spores.

Finally, the striking similarity between the appearance of the terraces and likely 2D nuclei in the multilayer inner coat and in inorganic and macromolecular crystals suggest that at least this part of the coat may assemble by crystallization mechanisms. A consequence of a crystallization spore coat assembly mechanism is that coat structure will be influenced by conditions during which these proteins self-assemble. In particular, variations in rates of 2D nucleation on spores could change the growth rate and hence the thickness of the spore coat, and this could influence spore properties such as their resistance and germination. The challenge now will be to correlate spore coat features identified in this work with specific coat proteins, and to understand how individual proteins contribute to these coat features, in particular, by using AFM-based immunolabeling techniques [29].

## References

[1] Klobutcher LA, Ragkousi K, Setlow P (2006) The *Bacillus subtilis* spore coat provides “eat resistance” during phagocytic predation by the protozoan *Tetrahymena thermophila* . Proc Natl Acad Sci USA 103: 165–70. 16371471 10.1073/pnas.0507121102 PMC1324984

[2] Henriques AO, Moran CP Jr (2007) Structure, assembly, and function of the spore surface layers. Annu Rev Microbiol 61: 555–588. 18035610 10.1146/annurev.micro.61.080706.093224

[3] Laaberki MH, Dworkin J (2008) Role of spore coat proteins in the resistance of *Bacillus subtilis* spores to *Caenorhabditis elegans* predation. J Bacteriol 190: 6197–6203. 18586932 10.1128/JB.00623-08 PMC2546783

[4] McKenney PT, Driks A, Eichenberger P (2013) The *Bacillus subtilis* endospore: assembly and functions of the multilayered coat. Nature Rev Microbiol 11: 33–44. 23202530 10.1038/nrmicro2921 PMC9910062

[5] McKenney PT, Eichenberger P (2012) Dynamics of spore coat morphogenesis in *Bacillus subtilis* . Mol Microbiol 83: 245–260. 22171814 10.1111/j.1365-2958.2011.07936.x PMC3256263

[6] de Hoon MJL, Eichenberger P, Vitkup D (2010) Hierarchical evolution of the bacterial sporulation network. Curr Biol 20: 735–745. 10.1016/j.cub.2010.06.031 PMC2944226 20833318

[7] McKenney PT, Driks A, Eskandarian HA, Grabowski P, Guberman J, et al (2010) A distance-weighted interaction map reveals a previously uncharacterized layer of the *Bacillus subtilis* spore coat. Curr Biol 20: 934–938. 20451384 10.1016/j.cub.2010.03.060 PMC2920530

[8] Waller LN, Fox N, Fox KF, Fox A, Price RL (2004) Ruthenium red staining for ultrastructural visualization of a glycoprotein layer surrounding the spore of *Bacillus anthracis* and *Bacillus subtilis* . J Microbiol Meth 58: 23–30. 10.1016/j.mimet.2004.02.012 15177900

[9] Wang KH, Isidro AL, Domingues L, Eskandarian HA, McKenney PT, et al (2009) The coat morphogenetic protein SpoVID is necessary for spore encasement in *Bacillus subtilis* . Mol Microbiol 74: 634–649. 19775244 10.1111/j.1365-2958.2009.06886.x PMC2806667

[10] Beall BA, Driks A, Losick R, Moran CP Jr (1993) Cloning and characterization of a gene required for assembly of the *Bacillus subtilis* spore coat. J Bacteriol 175: 1705–1716. 8449878 10.1128/jb.175.6.1705-1716.1993 PMC203965

[11] Isticato R, Sirec T, Giglio R, Baccigalupi L, Pesce G, et al (2013) Flexibility of the programme of spore coat formation in *Bacillus subtilis*: bypass of CotE requirement by overproduction of CotH. PLoS One 8: e74949. 24086406 10.1371/journal.pone.0074949 PMC3785510

[12] Imamura D, Kuwana R, Takamatsu H, Watabe K (2010) Localization of proteins to different layers and regions of *Bacillus subtilis* spore coats. J Bacteriol 192: 518–524. 19933362 10.1128/JB.01103-09 PMC2805314

[13] Imamura D, Kuwana R, Takamatsu H, Watabe K (2011) Proteins involved in formation of the outermost layer of *Bacillus subtilis* spores. J Bacteriol 193: 4075–4080. 21665972 10.1128/JB.05310-11 PMC3147665

[14] Tang J, Krajcikova D, Zhu R, Ebner A, Cutting S, et al (2007) Atomic force microscopy imaging and single molecule recognition force spectroscopy of coat proteins on the surface of *Bacillus subtilis* spore. J Mol Recog 20: 483–489. 10.1002/jmr.828 17932994

[15] Abhyankar W, Ter Beek A, Dekker H, Kort R, Brul S, et al (2011) Gel-free proteomic identification of the *Bacillus subtilis* insoluble coat protein fraction. Proteomics 11: 4541–4550. 21905219 10.1002/pmic.201100003

[16] De Francesco M, Jacobs JZ, Nunes F, Serrano M, McKenney PT, et al (2012) Physical interactions between coat morphogenetic proteins SpoVID and CotE is necessary for spore encasement in *Bacillus subtilis* . J Bacteriol 194: 4941–4950. 22773792 10.1128/JB.00914-12 PMC3430338

[17] Kim H, Hahn M, Grabowski P, McPherson DC, Otte MM, et al (2006) The *Bacillus subtilis* spore coat protein interaction network. Mol Microbiol 59: 487–502. 16390444 10.1111/j.1365-2958.2005.04968.x

[18] Krajcikova D, Lukacova M, Mullerova D, Cutting SM, Barak I (2009) Searching for protein-protein interactions within the *Bacillus subtilis* spore coat. J Bacteriol 191: 3212–3219. 19304857 10.1128/JB.01807-08 PMC2687167

[19] Mullerova D, Krajcikova D, Barak I (2009) Interactions between *Bacillus subtilis* early spore coat morphogenetic proteins. FEMS Microbiol Lett 299: 74–85. 19702880 10.1111/j.1574-6968.2009.01737.x

[20] Qiao H, Krajcikova D, Xing C, Lu B, Hao J, et al (2013) Study of the interactions between the key spore coat morphogenetic proteins CotE and SpoVID. J Struct Biol 181: 128–135. 23178679 10.1016/j.jsb.2012.11.002

[21] Chada VGR, Sanstad EA, Wang R, Driks A (2003) Morphogenesis of *Bacillus* spore surfaces. J Bacteriol 185: 6255–6261. 14563859 10.1128/JB.185.21.6255-6261.2003 PMC219407

[22] Plomp M, Leighton TJ, Wheeler KE, Malkin AJ (2005) The high-resolution architecture and structural dynamics of *Bacillus* spores. Biophys J 88: 603–608. 15501940 10.1529/biophysj.104.049312 PMC1305037

[23] Plomp M, Leighton TJ, Wheeler KE, Malkin AJ (2005) Architecture and high-resolution structure of *Bacillus thuringiensis* and *Bacillus cereus* spore coat surfaces. Langmuir 21: 7892–7898. 16089397 10.1021/la050412r

[24] Plomp M, Leighton TJ, Wheeler KE, Pitesky ME, Malkin AJ (2005) *Bacillus atrophaeus* outer spore coat assembly and ultrastructure. Langmuir 21: 10710–10716. 16262341 10.1021/la0517437

[25] Plomp M, Leighton TJ, Wheeler KE, Hill HD, Malkin AJ (2007) *In vitro* high-resolution structural dynamics of single germinating bacterial spores. Proc Nac Acad Sci USA 104: 9644–9649. 10.1073/pnas.0610626104 PMC1877984 17535925

[26] McPherson DC, Kim H, Hahn M, Wang R, Grabowski P, et al (2005) Characterization of the *Bacillus subtilis* spore morphogenetic coat protein CotO. J Bacteriol 187: 8278–8290. 16321932 10.1128/JB.187.24.8278-8290.2005 PMC1317010

[27] Carroll AM, Plomp M, Malkin AJ, Setlow P (2008) Protozoal digestion of coat-defective *Bacillus subtilis* spores produces “rinds” composed of insoluble coat protein. Appl Environ Microbiol 74: 5875–5881. 18689521 10.1128/AEM.01228-08 PMC2565959

[28] Ghosh S, Setlow B, Wahome PG, Cowan AE, Plomp M, et al (2008) Characterization of spores of *Bacillus subtilis* that lack most coat layers. J Bacteriol 190: 6741–6748. 18723620 10.1128/JB.00896-08 PMC2566211

[29] Plomp M, Malkin AJ (2009) Mapping of proteomic composition on the surfaces of *Bacillus* spores by atomic force microscopy. Langmuir 25: 403–409. 19063625 10.1021/la803129r

[30] Malkin AJ, Plomp M (2010) High-resolution architecture and structural dynamics of microbial and cellular system: Insights from high-resolution *in vitro* atomic force microscopy. In: Kalinin SV, Gruverman A, editors. Scanning probe microscopy of functional materials: nanoscale imaging and spectroscopy. New York: Springer. pp. 39–68.

[31] Plomp M, McCaffery JM, Cheong I, Huang X, Bettegowda C, et al (2007) Spore coat architecture of *Clostridium novyi* NT spores. J Bacteriol 189: 6457–6468. 17586633 10.1128/JB.00757-07 PMC1951917

[32] Anagnostopoulos C, Spizizen J (1961) Requirements for transformation in *Bacillus subtilis* . J Bacteriol 81: 741–746. 16561900 10.1128/jb.81.5.741-746.1961 PMC279084

[33] Maniatis T, Fritsch EF, Sambrook J (1982) Molecular cloning: A laboratory manual. Cold Spring Harbor, NY: Cold Spring Harbor Laboratory. 545 p.

[34] Nicholson WL, Setlow P (1990) Sporulation, germination and outgrowth. In: Harwood CR, Cutting SM, editors. Molecular biological methods for *Bacillus*. Chichester, UK: JohnWiley & Sons Ltd. pp. 391–450.

[35] Ragkousi K, Setlow P (2004) Transglutaminase-mediated cross-linking of GerQ in the coats of *Bacillus subtilis* spores. J Bacteriol 186: 5567–75. 15317760 10.1128/JB.186.17.5567-5575.2004 PMC516844

[36] Monroe A, Setlow P (2006) Localization of the transglutaminase cross-linking sites in the *Bacillus subtilis* spore coat protein GerQ. J Bacteriol 188: 7609–16. 16936016 10.1128/JB.01116-06 PMC1636287

[37] Westphal AJ, Price PB, Leighton TJ, Wheeler KE (2003) Kinetics of size changes of individual *Bacillus thuringiensis* spores in response to changes in relative humidity. Proc Natl Acad Sci USA 100: 3461–3466. 12584363 10.1073/pnas.232710999 PMC152315

[38] Driks A (2003) The dynamic spore. Proc Natl Acad Sci USA 100: 3007–3009. 12631692 10.1073/pnas.0730807100 PMC152230

[39] Aronson AI, Fitz-James P (1976) Structure and morphogenesis of the bacterial spore coat. Bact Rev 40: 360–402. 786255 10.1128/br.40.2.360-402.1976 PMC413961

[40] Holt SC, Leadbetter ER (1969) Comparative ultrastructure of selected aerobic spore-forming bacteria: a freeze etching study. Bacteriol Rev 33: 346–378. 4979698 10.1128/br.33.2.346-378.1969 PMC378324

[41] Wehrli E, Scherrer P, Kübler O (1980) The crystalline layers in spores of *Bacillus cereus* and *Bacillus thuringiensis* studied by freeze-etching and high resolution electron microscopy. Eur J Cell Biol 20: 283–289. 6766865

[42] Dufrêne YF, Boonaert CJP, Gerin PA, Asther M, Rouxhet PG (1999) Direct probing of the surface ultrastructure and molecular interactions of dormant and germinating spores of *Phanerochaete chrysosporium* . J Bacteriol 181: 5350–5354. 10464206 10.1128/jb.181.17.5350-5354.1999 PMC94041

[43] Dufrêne YF (2004) Using nanotechnologies to explore microbial surfaces. Nature Rev Microbiol 2: 451–458. 15152201 10.1038/nrmicro905

[44] Henriques AO, Moran CP Jr (2000) Structure and assembly of the bacterial endospore coat. Methods 20: 95–110. 10610808 10.1006/meth.1999.0909

[45] Takamatsu H, Kodama T, Nakayama T, Watabe K (1999) Characterization of the *yrbA* gene of *Bacillus subtilis*, involved in resistance and germination of spores. J Bacteriol 181: 4986–4994. 10438771 10.1128/jb.181.16.4986-4994.1999 PMC93988

[46] Zilhão R, Naclerio G, Baccigalupi L, Henriques AO, Moran CP Jr, et al (1999) Assembly requirements and role of CotH during spore coat formation in *Bacillus subtilis* . J Bacteriol 181: 2631–2633. 10198031 10.1128/jb.181.8.2631-2633.1999 PMC93693

[47] Maiwa K, Plomp M, van Enckevort WJP, Bennema P (1998) AFM observation of barium nitrate {111} and {100} faces: spiral growth and two-dimensional nucleation growth. J Cryst Growth 186: 214–223.

[48] Rogilo DI, Fedina LI, Kosolobov SS, Ranguelov BS, Latyshev AV (2013) Critical terrace width for two-dimensional nucleation during Si growth on Si (111)-(7×7) surface. Phys Rev Lett 111: 036105. 23909343 10.1103/PhysRevLett.111.036105

[49] Malkin AJ, Kuznetsov YuG, Land TA, DeYoreo JJ, McPherson A (1995) Mechanisms of growth for protein and virus crystals. Nature Struct Biol 2: 956–959. 7583668 10.1038/nsb1195-956

[50] Malkin AJ, Kuznetsov YuG, McPherson A (1999) *In situ* atomic force microscopy studies of surface morphology, growth kinetics, defect structure and dissolution in macromolecular crystallization. J Cryst Growth 196: 471–488.

[51] Malkin AJ, McPherson A (2004) Probing of crystal interfaces and the structures and dynamic properties of large macromolecular ensembles with in situ atomic force microscopy. In: Lin XY, DeYoreo JJ, editors. From solid-liquid interface to nanostructure engineering, vol. 2. New York: Plenum/Kluwer Academic Publisher. pp. 201–208.

[52] DeYoreo JJ, Vekilov PG (2003) Principles of crystal nucleation and growth. In: Dove PM, DeYoreo JJ, Weiner S, editors. Biomineralization. Washington, DC: Mineral Society of America. pp. 57–93.

[53] Plomp M, McPherson A, Larson SB, Malkin AJ (2001) Growth mechanisms and kinetics of trypsin crystallization. J Phys Chem B 105: 542–551.

[54] Kuznetsov YuG, Malkin AJ, Land TA, DeYoreo JJ, Barba AP, et al (1997) Molecular resolution imaging of macromolecular crystals by atomic force microscopy. Biophys J 72: 2357–2364. 9129839 10.1016/S0006-3495(97)78880-5 PMC1184431

[55] Moir A (1981) Germination properties of a spore coat-defective mutant of *Bacillus subtilis* . J. Bacteriol 146: 1106–1116. 6787012 10.1128/jb.146.3.1106-1116.1981 PMC216967

[56] Qiu X, Setlow P (2009) Structural and genetic analysis of X-ray scattering by spores of *Bacillus subtilis* . J Bacteriol 191: 7620–7622. 19837800 10.1128/JB.01200-09 PMC2786606

[57] Sousa JCF, Silva MT, Balassa G (1976) Exosporium-like outer layer in *Bacillus subtilis* spores. Nature 263: 53–54. 822351 10.1038/263053a0

[58] Kaillas L, Terry C, Abbott N, Taylor R, Mullin N, et al (2011) Surface architecture of endospores of the *Bacillus cereus*/*anthracis*/*thuringiensis* family at the subnanometer scale. Proc Natl Acad Sci USA 108: 16014–16019. 21896762 10.1073/pnas.1109419108 PMC3179049

[59] Gebbink MF, Claessen D, Bouma B, Dijkhuizen L, Wösten HA (2005) Amyloids – a functional coat for microorganisms. Nature Rev Microbiol 3: 333–341. 15806095 10.1038/nrmicro1127

[60] Hashimoto T, Wu-Yuan CD, Blumenthal HJ (1976) Isolation and characterization of the rodlet layer of *Trichophyton mentagrophytes* microconidial wall. J Bacteriol 127: 1543–1549. 956129 10.1128/jb.127.3.1543-1549.1976 PMC232951

[61] Beever RE, Redgewell RJ, Dempsey G (1979) Purification and chemical characterization of the rodlet layer of *Neurospora crassa* conidia. J Bacteriol 140: 1063–1070. 160407 10.1128/jb.140.3.1063-1070.1979 PMC216753

[62] Wessels JGH (1998) Hydrophobins: Proteins that change the nature of the fungal surface. Adv Microbial Physiol 38: 1–45. 10.1016/s0065-2911(08)60154-x 8922117

[63] Wösten HAB, de Vocht ML (2000) Hydrophobins, the fungal coat unravelled. Biochim Biophys Acta 1469: 79–86. 10998570 10.1016/s0304-4157(00)00002-2

[64] Claessen D, Stokroos I, Deelstra HJ, Penninga NA, Bormann C, et al (2004) The formation of the rodlet layer of streptomycetes is the result of the interplay between rodlins and chaplins. Mol Microbiol 53: 433–443. 15228525 10.1111/j.1365-2958.2004.04143.x

[65] Dobson CM (2003) Protein folding and misfolding. Nature 426: 884–890. 14685248 10.1038/nature02261

[66] Smith JF, Knowles TPJ, Dobson CM, MacPhee CE, Welland ME (2006) Characterization of the nanoscale properties of individual amyloid fibrils. Proc Natl Acad Sci USA 103: 15806–15811. 17038504 10.1073/pnas.0604035103 PMC1635084

[67] Isticato R, Cangiano G, Tran HT, Ciabattini A, Medaglini D, et al (2001) Surface display of recombinant proteins on *Bacillus subtilis* spores. J Bacteriol 183: 6294–6301. 11591673 10.1128/JB.183.21.6294-6301.2001 PMC100119

[68] Driks A (1999) *Bacillus subtilis* spore coat. Microbiol Mol Bio Rev 63: 1–20. 10066829 10.1128/mmbr.63.1.1-20.1999 PMC98955

[69] Zheng LB, Donovan WP, Fitz-James PC, Losick R (1988) Gene encoding a morphogenic protein required in the assembly of the outer coat of the *Bacillus subtilis* endospore. Genes Dev 2: 1047–1054. 3139490 10.1101/gad.2.8.1047

[70] Zilhão R, Serrano M, Isticato R, Ricca E, Moran CP Jr, et al (2004) Interactions among CotB, CotG, and CotH during assembly of the *Bacillus subtilis* spore coat. J Bacteriol 186: 1110–1119. 14762006 10.1128/JB.186.4.1110-1119.2004 PMC344205

[71] Donovan W, Zheng LB, Sandman K, Losick R (1987) Genes encoding spore coat polypeptides from *Bacillus subtilis* . J Mol Biol 196: 1–10. 2821284 10.1016/0022-2836(87)90506-7

[72] Naclerio G, Baccigalupi L, Zilhao R, de Felice M, Ricca E (1996) *Bacillus subtilis* spore coat assembly requires *cotH* gene expression. J Bacteriol 178: 4375–4380. 8755863 10.1128/jb.178.15.4375-4380.1996 PMC178202

[73] Ozin AJ, Henriques AO, Yi H, Moran CP Jr (2000) Morphogenetic proteins SpoVID and SafA form a complex during assembly of the *Bacillus subtilis* spore coat. J Bacteriol 1828–1833. 10714986 10.1128/jb.182.7.1828-1833.2000 PMC101864

[74] Frenkel D (2006) Physical chemistry - Seeds of phase change. Nature 443: 641–641. 17035987 10.1038/443641a

[75] Kuznetsov YuG, Malkin AJ, McPherson A (1997) Atomic force microscopy studies of living cells: Visualization of motility, division, aggregation, transformation and apoptosis. J Struct Biol 120: 180–191. 9417982 10.1006/jsbi.1997.3936

[76] Magonov SN, Elings V, Whangbo MH (1997) Phase imaging and stiffness in tapping-mode atomic force microscopy. Surf Sci 375: L385–L391.

[77] Magonov SN, Cleveland J, Elings V, Denley D, Whangbo M-H (1997) Tapping-mode atomic force microscopy study of the near-surface composition of a styrene-butadiene-styrene triblock copolymer film. Surf Sci 389: 201–211.

[78] Butzin XY, Troiano AJ, Coleman WH, Griffiths KK, Doona CJ, et al (2012) Analysis of the effects of a *gerP* mutation on the germination of spores of *Bacillus subtilis* . J Bacteriol 194: 5749–5758. 22904285 10.1128/JB.01276-12 PMC3486119

[79] Chernov AA (1984) Modern crystallography. III. Crystal growth. Berlin: Springer-Verlag. 517 p.

[80] Cabrera N, Vermilyea DA (1958) The growth of crystals from solution. In: Doremus RH, Roberts BW, Turnbul D, editors. Growth and perfection of crystals. New York: Wiley. pp. 393–410.

[81] van Enckevort WJP, van der Berg ACJF, Kreuwel KBG, Derksen AJ, Couto MS (1996) Impurity blocking of growth steps: experiments and theory. J Cryst Growth 166: 156–161.

[82] Land TA, Martin TL, Potapenko S, Palmore GT, DeYoreo JJ (1999) Recovery of surfaces from impurity poisoning during crystal growth. Nature 399: 442–445.

[83] Plomp M, McPherson A, Malkin AJ (2003) Repair of impurity-poisoned protein crystal surfaces. Proteins: Struct, Function, Bioinform 50: 486–495. 10.1002/prot.10288 12557190

[84] Driks A (2002) Maximum shields: the assembly and function of the bacterial spore coat. Trends Microbiol 10: 251–254. 12088650 10.1016/s0966-842x(02)02373-9

[85] Hollander FFA, Plomp M, van de Streek CJ, van Enckevort WJP (2001) A two-dimensional Hartman-Perdok analysis of polymorphic fat surfaces observed with atomic force microscopy. Surf Sci 471: 101–113.

[86] Plomp M, van Enckevort MJV, van Hoof PJCM, van de Streek CJ (2003) Morphology and dislocation movement in n-C_40_H_82_ paraffin crystals grown from solution. J Cryst Growth 249: 600–613.

[87] Eichenberger P, Jensen ST, Conlon EM, van Ooij C, Silvaggi J, et al (2003) The σ^E^ regulon and the identification of additional sporulation genes in *Bacillus subtilis* . J Mol Biol 327: 945–972. 12662922 10.1016/s0022-2836(03)00205-5

